# Influence of magnetic field on MHD mixed convection in lid-driven cavity with heated wavy bottom surface

**DOI:** 10.1038/s41598-023-45707-x

**Published:** 2023-11-02

**Authors:** Mst. Umme Mahmuda Maya, Md. Nur Alam, Ahmed Refaie Ali

**Affiliations:** 1 Department of Mathematics, Pabna University of Science and Technology, Pabna, 6600 Bangladesh; 2 Department of Mathematics and Computer Science, Faculty of Science, Menoufia University, Shebin El Kom, 32511 Menofia Governorate Egypt

**Keywords:** Fluid dynamics, Applied mathematics

## Abstract

This study investigates the influence of a rectangular heat source on magnetohydrodynamic hybrid convection flow in a lid-driven cavity. The effects of various parameters, such as the heat source size, magnetic field strength, and heat absorption/generation, are analyzed. The results show that increasing the heat source size decreases the average Nusselt number along the heated wall. The average Nusselt number also decreases with higher magnetic field strength and heat generation, while it increases with heat absorption. The major finding is to apply an important technique the Galerkin weighted residual technique of the finite element (FE) method to solve the non-dimensional equations and the associated boundary conditions. The isotherms are used to show the temperature distribution in a domain. Streamline present the flow field in the enclosure. However, it is easy to realize the direction and intensity of the heat transfer particularly in convection problems which the path of heat flux is perpendicular and the isotherm due to convection effect. Thus, the purpose of this research is to study the results of mixed convection. The effects of location and height of the partitions are considered for the various Richardson numbers. Fluid flow field, thermal field and heat transfer are presented through the streamlines and isotherms, respectively. Results are substantiated relating to the published work.

## Introduction

In many studies, the influence of a closed space rectangular heat source on MHD hybrid convection flow into the lid-driven cavity, keeping a heated waved bottom surface with internal heat absorption or generation is an important problem in applied mathematics, physics and engineering. Applications of the considered geometry can be seen in geophysical problems, thermal hydraulics of nuclear reactors, solar collectors, float glass manufacturing, crystal growth, paper production, heat exchangers, grain storage, wire drawing, petroleum exploration, chemical engineering, mechanical engineering and nuclear reactor and so on. A convection system applying both forced and free convection is remarkably known as mixed convection^1–7^. Free convection is an approach to heat transfer where an external source does not generate fluid motion. Instead, the liquid motion is caused by the buoyancy, the difference in fluid density due to the temperature gradient. The most common example of natural convection is sea and land breeze. Forced convection stands for a process or a type of carriage in which the external source creates fluid motion. Examples of forced convection are fan, and pump suction device. Many industrial applications, including residential ventilation, electronic cooling appliances^9–11^, food processing^13–15^, Neural Networks Optimization^16–23^ and solar collectors^6–8^, dynamics of lakes and reservoirs, thermal hydraulics of nuclear reactors, float glass manufacturing, crystal growth, paper production, wire drawing, petroleum exploration, chemical engineering, mechanical engineering as well the boundary layer management in the area of aerodynamics require combined convection heat transference and flow into a lid-driven chamber.

Over the earlier three decades, considerable experimental and numerical analyses contain been in the literature on flow and heat transference into the lid-driven chamber. Combined free convection of non-Newtonian hybrid nanoparticles was recently investigated by Hussain et al*.*^8^ inside the wavy-shaped cavity. Their statistical investigations indicate that the optimal rate of heat transfer for pseudo-plastic hybrid nano-liquid was achieved with an increased Rayleigh number, heat conductivity ratio, and a low Hartman number. Hamzah et al*.*^9^ investigated magneto-hydrodynamics combined convection and entropy creation of CNT-water nanoparticles in a porous lid-driven waved chamber with distinct boundary circumstances. Asad et al.^10,11^ analyzed free convection flow heat transfer with vertical wavy walls inside the enclosure. Saha et al*.*^12^ examined the hybrid convection flows and heat transference within a lid-driven chamber with a waved base surface. It has been scrutinised that the waved lid-driven chamber is assumed to have an efficacious heat transference mechanism with greater waved surface amplitudes and higher Grashofs number. Chabani et al.^13^ recently inquired the magneto-hydrodynamic flow conjugate nano-fluid into a triangular section with an elliptical enclosure. Ahmed et al.^14^ studied heat transmission in a porous medium and nanofluid-filled complicated undulating enclosure with periodic temperature. They informed that heat transmission increments as the volume fraction of nanofluids grows. As the interior heat absorption parameter and number of cavity undulations increases, the wavy surface amplitude decreases. Furthermore, decreased internal heat generation/absorption parameters increase horizontal and vertical velocities. Raizah et al.^15^ investigated magneto-hydrodynamics hybrid convection of conjugate nanofluid in a alveolar waved cavity utilizing local fervent non-equilibrium circumstances. Oglakkaya et al.^16^ studied the flow of irregular MHD hybrid convection within a lid-driven waved enclosure with a heated surface. Barman et al*.*^17^ carried out the free convection within a free convection within an insulated barrier with a heat source attached to porous enclosure. They claimed that the mathematical correlation and mean Nusselt number with the controlling parameters are offered to compute the heater's maximum temperature. Asad et al.^18,19^ studied the impact of fin length on convection and heat transport in a wavy enclosure. Elatar et al.^20^ analyzed laminar free convective heat inside a square enclosure by attaching an irregular horizontal fin to a heated wall at various lengths and places. The impression of fin location and frame length on flow characteristics and heat-reduction components has been researched. Hussain et al.^21^ studied the fins and bended magnetic field influence in a twice lid-driven chamber with nanofluid filled with Cu–water. The impression of parallel insulated baffles with open enclosures was studied by Palaniappan et al*.*^22^. The impression of fin thickness on the hybrid convection circulation of a conjugate nanofluid in the existence of a magnetic field with an efficient heat sink was investigated by Shorbagy et al.^23^. They claimed that the Richardson number changed at various fin thicknesses. The effect of fin thickness on the hybrid convection circulation of a conjugate nanofluid in the existence of a magnetic field with an efficient heat sink was investigated by Bakar et al.^6^. They observed that the Richardson number changed at various fin thicknesses. Chamkha et al.^7^ investigated unstable laminar combined convective flow and heat transmission of a conductive fluid and heat production or absorption fluid with a plumb lid-driven chamber in the availability of the magnetic field. According to their findings, the magnetic field has a momentous effect on the flow behavior and heat transference characteristics within the chamber. The MHD mixed convection inside a Vented Cavity with Heat creation or Absorption in the appearance of a Cylindrical Obstacle was explored by Kazi et al*. *^8^. Saha et al*.*^24^ scrutinised internal heat-producing or absorbing effects on MHD combined convection flow inside lid-driven enclosures. They found that while the heat transference rate reduces with increments in the Hartmann (Ha) number and the heat creation parameter, it increases with the increments in the heat absorption parameter. Quyyum et al.^25^ studied magneto-hydrodynamic combined convection and heat-generating tangent hyperbolic nanofluid with Newtonian heat transfer. And other similarities can be found Johnson et al*.*^26^, Yang et al*.*^27^,Bouterra et al*.*^28^, and Laurel et al*.*^29^. In addition, the finite element (FE) method, magnetic field, mixed/hybrid convection, and heat source are more detailed^30–32^.

In all prior analyses, the convection flow and heat transference in the waved cavity is influenced by fluids that conduct electrically in magnetic fields, thermal boundaries, vertical fins, and heat creation or absorption. In all preceding investigations, the convective flow and heat transference in the waved cavity altered with electrically producing fluids within the appearance of magnetic fields, thermal barriers, vertical fins, and heat creation or absorption. Overall,all of the above research can be classified into three categories. The first set focuses on MHD mixed and natural convection^9–19^ within a square wavy cavity, the second set deals with MHD mixed and natural convection within a square wavy cavity having fins^6,20–23^, and the third on MHD mixed and natural convection with heat generation or absorption^7,8,24–29^. Effects of Electromagnetic fields on plasma and Hall currents on viscoelastic fluid have been studied^37–39^ with nonlinear heat and mass transfer. Refaie et al.^40^ examine soliton solutions in nonlinear Heisenberg ferromagnetic spin chains, while the works by Jawad et al.^41,42^ investigate nanofluid stagnation point flow with melting heat transfer. Ali J. Chamkha et al. studied some investigations of external effects such as Hall and ion slip effects on MHD Nanofluid in the presence of heat and mass transfer ^43–53^. Waqar Azeem Khan et al.^54–60^ investigated some studies on MHD Nanofluid subjected to physical phenomena.

To the best of the authors' sense, there is currently no existing study that has comprehensively examined the impact of an enclosed rectangular heat source on magnetohydrodynamic (MHD) mixed convection flow within a lid-driven cavity. This investigation specifically considers a scenario where the cavity features a heated undulating bottom surface that either generates or absorbs internal heat.

The exact novelty of the presented work is to apply an important technique the Galerkin weighted residual technique of the finite element (FE) method to solve the non-dimensional equations and the associated boundary conditions. Heatline technique is an important method to visualize heat transport in the influence of a closed space rectangular heat source on MHD hybrid convection flow into the lid-driven cavity, keeping a heated waved bottom surface with internal heat absorption or generation. The isotherms are used to show the temperature distribution in a domain. Streamline present the flow field in the enclosure. However, it is easy to realize the direction and intensity of the heat transfer particularly in convection problems which the path of heat flux is perpendicular and the isotherm due to convection effect. Thus, the purpose of this research is to study the results of mixed convection. The effects of location and height of the partitions are considered for the various Richardson numbers. Fluid flow field, thermal field and heat transfer are presented through the streamlines and isotherms, respectively.

## Problem formulation and governing equation

**Figure 1:**
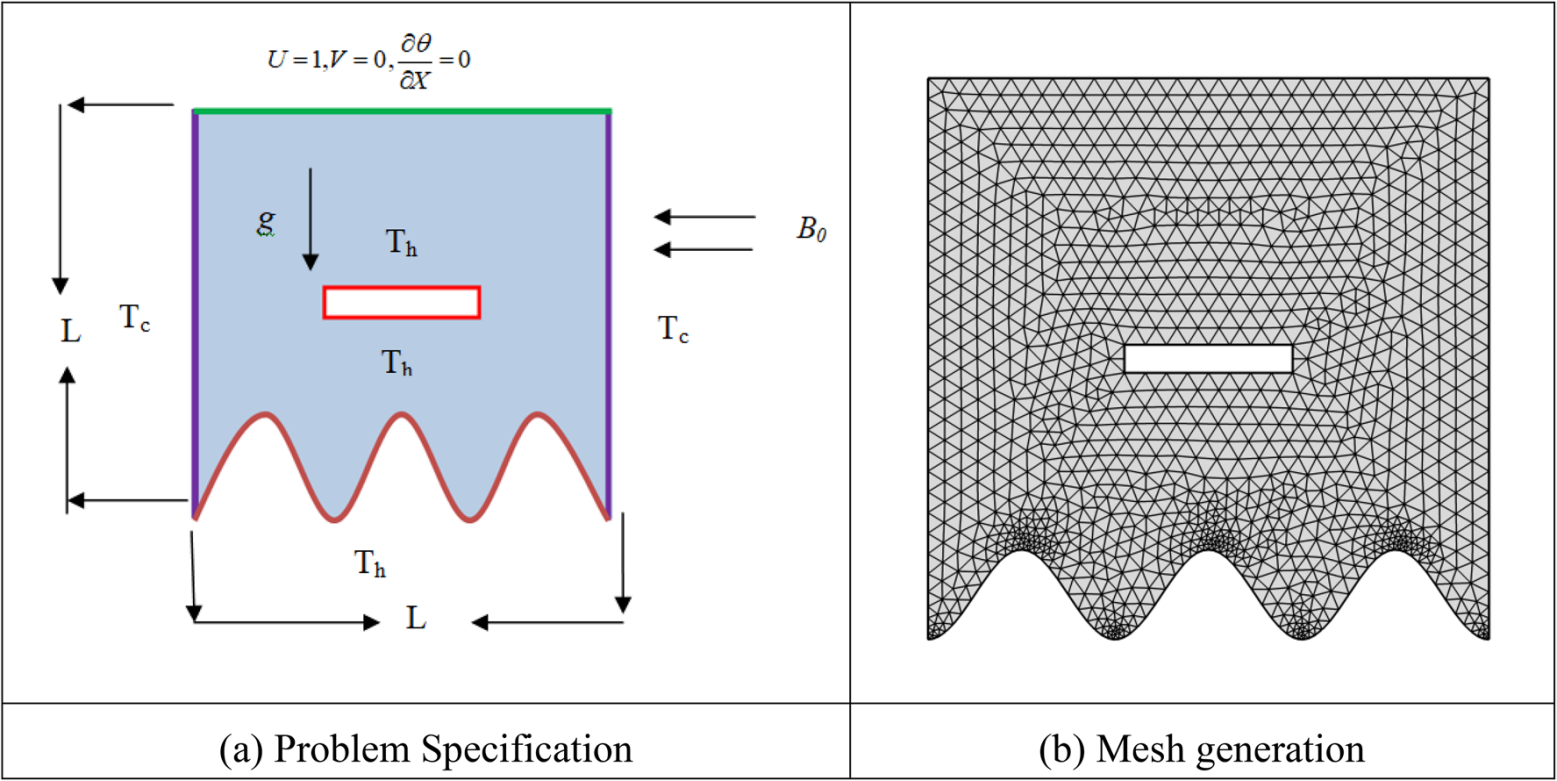
Problem configuration.

The physical parameters of the square waved enclosure considered for this inquiry are illustrated in Fig. 1. The peak wall of the undulating hollow is moving with a constant velocity of unity $U_{0}$, while the other walls are in no-slip conditions. The plumb walls of the square wavy section are kept at cold temperatures $Tc$*,* while the pick wall is isolated, and the closed space rectangular source of high temperature $T_{h}$ is installed in the cavity's center. The source's height is *H*, and the width is *W*. Additionally, the temperature on the bottom wall is retained at a greater scale $T_{h}$. Since it is supposed that the fluid is electrically conducting, the walls of the wave section are expected to be electrically insulating. In the x-direction, there is a uniform petition of a magnetic field with the same magnitude, *B0*, everywhere. When weighed against the magnetic field that is being applied, the initiated magnetic field that is created by the velocity of an electrically conducting (*EC*) fluid is often disregarded as being of little significance. With the density anomaly, which shifts according to the Boussinesq approximation^34^, the thermo-physical properties of liquids are thought to remain unchanged. Generally, it is occupied that the enclosing fluid has a Newtonian flow that is incompressible, steady, and laminar.

Following the primary hypotheses, the governing continuity, momentum, and energy equations may be formulated:

The continuity equation

1 $$\frac{\partial u}{{\partial x}} + \frac{\partial v}{{\partial y}} = 0$$

Momentum equations

2 $$u\frac{\partial u}{{\partial x}} + v\frac{\partial u}{{\partial y}} = - \frac{1}{\rho }\frac{\partial p}{{\partial x}} + \upsilon \nabla^{2} u$$

3 $$u\frac{\partial v}{{\partial x}} + v\frac{\partial v}{{\partial y}} = - \frac{1}{\rho }\frac{\partial p}{{\partial y}} + \upsilon \nabla^{2} v + g\beta \left( {T - T_{c} } \right) - \frac{{\sigma B_{0}^{2} v}}{\rho }$$

Energy equations

4 $$u\frac{\partial T}{{\partial x}} + v\frac{\partial T}{{\partial y}} = \alpha \nabla^{2} T + \frac{{Q_{0} }}{{\rho c_{p} }}\left( {T - T_{c} } \right)$$

where $u$ and $v$ is the variables of velocity of the fluid in the $x$ and $y$ directions, $p$ is the pressure, *x*, *y* is the coordinate directions, *T *is the temperature, *β* is fluid temperature expansion coefficient. The parameters *g*, *B*_0_ and $Q_{0}$ are, in order, the acceleration caused by gravity, the magnetic induction, and the coefficient of heat generation $\left( {Q_{0} > 0} \right)$ or absorption $\left( {Q_{0} < 0} \right)$ inside the object .

### Initial and boundary conditions

The following are the boundary conditions for the current problem:

On the peak wall:

5a $$u = 1,\,v = 0\,,\frac{\partial T}{{\partial x}} = 0\,\,\,;\,\,0 \le x \le 1\,\,,\,\,y = 1$$

On the left wall:

5b $$u = \,v = 0\,,\,\,T = 0\,\,\,;\,\,\,0 \le y \le 1\,\,,\,\,and\,\,x = 0$$

On the right wall:

5c $$u = \,v = 0\,,\,\,T = 0\,\,;\,\,\,0 \le y \le 1\,\,,\,\,and\,\,x = 1$$

On the bottom wall:

5d $$u = \,v = 0\,,\,\,T = 1\,\,;\,for\,\,0 \le x \le 1\,,\,y = 0,\,and\,A\left( {1 - \cos \left( {2\pi \lambda x} \right)} \right)$$

On the rectangular surface:

5e $$u = \,v = 0\,,\,\,T = 1\,\,\,$$

### Dimensional analysis

By assuming dimensionless variables,

6 $$X = \frac{x}{L}\,,Y = \frac{y}{L}\,,U = \frac{u}{{U_{0} }}\,,V = \frac{v}{{U_{0} }}\,,P = \frac{p}{{\rho U_{0}^{2} }}\,,\theta = \frac{{T - T_{c} }}{{T_{h} - T_{c} }}$$

Introducing the aforementioned dimensionless scales into G.E. (governing equations) yields the non-dimensional formulations of the following equations:

7 $$\frac{\partial U}{{\partial X}} + \frac{\partial V}{{\partial Y}} = 0$$

8 $$U\frac{\partial U}{{\partial X}} + V\frac{\partial U}{{\partial Y}} = - \frac{\partial P}{{\partial X}} + \frac{1}{{\text{Re}}}\nabla^{2} U$$

9 $$U\frac{\partial V}{{\partial X}} + V\frac{\partial V}{{\partial Y}} = - \frac{\partial P}{{\partial Y}} + \frac{1}{{\text{Re}}}\nabla^{2} V + \frac{Gr}{{{\text{Re}}^{2} }}\theta - \frac{{Ha^{2} }}{{\text{Re}}}V$$

10 $$U\frac{\partial \theta }{{\partial X}} + V\frac{\partial \theta }{{\partial Y}} = \frac{1}{{{\text{Re}} \,\Pr }}\nabla^{2} \theta + \frac{\Delta }{{{\text{Re}} \,\Pr }}\theta$$

The parameters in the preceding equations

$${\text{Re}} = \frac{{U_{0} L}}{\upsilon }$$, $$\Pr = \frac{\upsilon }{\alpha }$$, $$Ri = \frac{Gr}{{{\text{Re}}^{2} }},$$ $$Ha^{2} = \frac{{\sigma B_{0}^{2} L^{2} }}{\mu }$$,

11 $$Gr = \frac{{g\beta \Delta TL^{3} }}{{\upsilon^{2} }} \quad {\text{and}}\quad \Delta = \frac{{Q_{0} L^{2} }}{{\alpha \rho C_{p} }}$$

are respectively represent the dimensionless Reynolds (*Re*) number, Prandtl (*Pr*) number, Hartmann (*Ha*) number, Grashof (*Gr*) number and heat generation or absorption ($\Delta$) coefficient.

The transformed non-dimensional b. c. (boundary conditions) are as follows:

On the top wall:

12a $$U = 1,\,V = 0\,,\frac{\partial \theta }{{\partial X}} = 0\,\,\,;\,\,0 \le X \le 1\,\,,\,\,Y = 1$$

On the left wall:

12b $$U = \,V = 0\,,\,\,\theta = 0\,\,\,;\,\,\,0 \le Y \le 1\,\,,\,\,and\,\,X = 0$$

On the right wall:

12c $$U = \,V = 0\,,\,\,\theta = 0\,\,;\,\,\,0 \le Y \le 1\,\,,\,\,and\,\,X = 1$$

On the bottom wall:

12d $$U = \,V = 0\,,\,\,\theta = 1\,\,;\,0 \le X \le 1,\,Y = 0,\,and\,A\left( {1 - \cos \left( {2\pi \lambda x} \right)} \right)$$

On the rectangular surface:

12e $$U = \,V = 0\,,\,\,\theta = 1$$

The heat dismissal via conduction is compared with heat dismissal due to the convection is developed: $h * \Delta T = - k\frac{\partial T}{{\partial n}}$. Where n is the surface normal .Local and average Nusselt number on the heated enclosure is defined as

13 $$Nu_{L} = \frac{\partial \theta }{{\partial N}}\left| {_{S} } \right.\quad {\text{and}}\quad Nu_{av} = \int\limits_{0}^{L} {\frac{\partial \theta }{{\partial N}}} \left| {_{S} } \right.ds$$

## Numerical procedure

The nonlinear governing equations, i.e., mass, momentum and energy conservation equations are transformed into a system of integral equations by using the Galerkin weighted residual method of finite-element formulation. The nonlinear algebraic equations so obtained are modified by imposition of boundary conditions. These modified nonlinear equations are transferred into linear algebraic equations with the help of Newton’s method. Lastly, these linear equations are solved by applying Triangular factorization. For numerical computation and post processing, the software COMSOL Multiphysics is used.

The Galerkin weighted residual technique of the finite element (FE) method is utilized to solve the non-dimensional Eqs. (7–10) and the associated boundary conditions (12a–12e). Taylor and Hood^34^ and Dechaumphai^35^ provide thorough explanations of the development of this method and the computational process involved. To solve Eqs. (12)–(13), we use the penalty finite element approach, in which the pressure is abolished by a penalty parameter $\gamma$ while the incompressibility criterion provided by Eq. (1) (ref.^34^) resulting in

14 $$P = - \gamma \left( {\frac{\partial U}{{\partial X}} + \frac{\partial V}{{\partial Y}}} \right)$$

For sufficiently high quantities of $\gamma$, the continuity Eq. (11) can be manually verified. Using Eq. (20), the momentum Eqs. (12) and (13) reduce to

15 $$U\frac{\partial U}{{\partial X}} + V\frac{\partial V}{{\partial Y}} = \gamma \frac{\partial }{\partial X}\left( {\frac{\partial U}{{\partial X}} + \frac{\partial V}{{\partial Y}}} \right) + \frac{1}{{\text{Re}}}\nabla^{2} U$$

16 $$U\frac{\partial V}{{\partial X}} + V\frac{\partial V}{{\partial Y}} = \gamma \frac{\partial }{\partial Y}\left( {\frac{\partial U}{{\partial X}} + \frac{\partial V}{{\partial Y}}} \right) + \frac{1}{{\text{Re}}}\nabla^{2} V + \frac{Gr}{{{\text{Re}}^{2} }}\theta - \frac{{Ha^{2} }}{{\text{Re}}}V$$

The GWR (Galerkin weighted residual) finite element (*FE*) method is used in order to numerically solve the governing dimensionless Eqs. (7)–(10) as well as the dimensionless boundary conditions (12a)–(12d).

Expanding the velocity components $\left( {U\,,V} \right)$ and temperature $\left( \theta \right)$ using basis set $\left\{ {\delta_{k} } \right\}_{k = 1}^{N}$ as,

17 $$U \approx \sum\nolimits_{k = 1}^{N} {U_{k} } \delta_{k} \left( {X,Y} \right),\quad V \approx \sum\nolimits_{k = 1}^{N} {V_{k} } \delta_{k} \left( {X,Y} \right),\quad \theta \approx \sum\nolimits_{k = 1}^{N} {\theta_{k} } \delta_{k} \left( {X,Y} \right)$$

At the nodes of the internal domain $\Omega$, the Galerkin finite element technique produces the following non-linear residual equations for Eqs. (15) and (16), respectively, into (10).

18 $$\begin{aligned} R_{i}^{1} & = \sum\limits_{k = 1}^{N} {U_{k} } \int_{\Omega } {\left[ {\left( {\sum\limits_{k = 1}^{N} {U_{k} \delta_{k} } } \right)\frac{{\partial \delta_{k} }}{\partial X} + \left( {\sum\limits_{k = 1}^{N} {V_{k} \delta_{k} } } \right)\frac{{\partial \delta_{k} }}{\partial Y}} \right]} \delta_{i} dXdY \\ &\quad+ \gamma \left[ {\sum\limits_{k = 1}^{N} {U_{k} } \int\limits_{\Omega } {\frac{{\partial \delta_{i} }}{\partial X}} \frac{{\partial \delta_{k} }}{\partial X}dXdY + \sum\limits_{k = 1}^{N} {V_{k} } \int\limits_{\Omega } {\frac{{\partial \delta_{i} }}{\partial X}} \frac{{\partial \delta_{k} }}{\partial Y}dXdY} \right] + \frac{1}{{\text{Re}}}\left[ \sum\limits_{k = 1}^{N} {U_{k} } \int\limits_{\Omega } {\left[ {\frac{{\partial \delta_{i} }}{\partial X}\frac{{\partial \delta_{k} }}{\partial X} + \frac{{\partial \delta_{i} }}{\partial Y}\frac{{\partial \delta_{k} }}{\partial Y}} \right]dXdY}\right] \end{aligned}$$

19 $$\begin{aligned} R_{i}^{2} & = \sum\limits_{k = 1}^{N} {V_{k} } \int_{\Omega } {\left[ {\left( {\sum\limits_{k = 1}^{N} {U_{k} \delta_{k} } } \right)\frac{{\partial \delta_{k} }}{\partial X} + \left( {\sum\limits_{k = 1}^{N} {V_{k} \delta_{k} } } \right)\frac{{\partial \delta_{k} }}{\partial Y}} \right]} \delta_{i} dXdY \\ &\quad+ \gamma \left[ {\sum\limits_{k = 1}^{N} {U_{k} } \int\limits_{\Omega } {\frac{{\partial \delta_{i} }}{\partial Y}} \frac{{\partial \delta_{k} }}{\partial X}dXdY + \sum\limits_{k = 1}^{N} {V_{k} } \int\limits_{\Omega } {\frac{{\partial \delta_{i} }}{\partial Y}} \frac{{\partial \delta_{k} }}{\partial Y}dXdY} \right] + \frac{1}{{\text{Re}}} \sum\limits_{k = 1}^{N} {V_{k} } \int\limits_{\Omega } {\left[ {\frac{{\partial \delta_{i} }}{\partial X}\frac{{\partial \delta_{k} }}{\partial X} + \frac{{\partial \delta_{i} }}{\partial Y}\frac{{\partial \delta_{k} }}{\partial Y}} \right]dXdY + \frac{Gr}{{{\text{Re}}^{2} }}\int\limits_{\Omega } {\left( {\sum\limits_{k = 1}^{N} {\theta_{k} \delta_{k} } } \right)} } \delta_{i} dXdY - \frac{{Ha^{2} }}{{\text{Re}}}\int\limits_{\Omega } {\left( {\sum\limits_{k = 1}^{N} {V_{k} \delta_{k} } } \right)\delta_{i} dXdY} \end{aligned}$$

20 $$\begin{aligned} R_{i}^{3} & = \sum\limits_{k = 1}^{N} {\theta_{k} } \int_{\Omega } {\left[ {\left( {\sum\limits_{k = 1}^{N} {U_{k} \delta_{k} } } \right)\frac{{\partial \delta_{k} }}{\partial X} + \left( {\sum\limits_{k = 1}^{N} {V_{k} \delta_{k} } } \right)\frac{{\partial \delta_{k} }}{\partial Y}} \right]} \delta_{i} dXdY\\ &\quad + \frac{1}{{{\text{Re}} \Pr }}\sum\limits_{k = 1}^{N} {\theta_{k} } \left[ {\int\limits_{\Omega } {\frac{{\partial \delta_{i} }}{\partial X}} \frac{{\partial \delta_{k} }}{\partial X} + \frac{{\partial \delta_{i} }}{\partial Y}\frac{{\partial \delta_{k} }}{\partial Y}} \right]dXdY + \frac{\Delta }{{{\text{Re}} \Pr }}\int\limits_{\Omega } {\left( {\sum\limits_{k = 1}^{N} {\theta_{k} \delta_{k} } } \right)\delta_{i} dXdY} \end{aligned}$$

where *N*, *i* and *k* represent the iteration number, the residual, and the number of nodes, respectively. Gaussian quadrature was used to distribute the following operations from Eqs. (18)-(20). Lastly, these equations are formed into a matrix form, which is then solved using Newton Raphson's iteration approach. Those interested may find more information in the previously announced activities^35,36^.

The convergence criterion of this method is imposes as

21 $$\left[ {\frac{{\psi^{n + 1} - \psi^{n} }}{{\psi^{n + 1} }}} \right] \le 10^{ - 5}$$

where, *ψ* represent $U\,,V,\theta$ as dependent variable and $n$ is a iteration number.

## Grid refinement test

**Figure 2:**
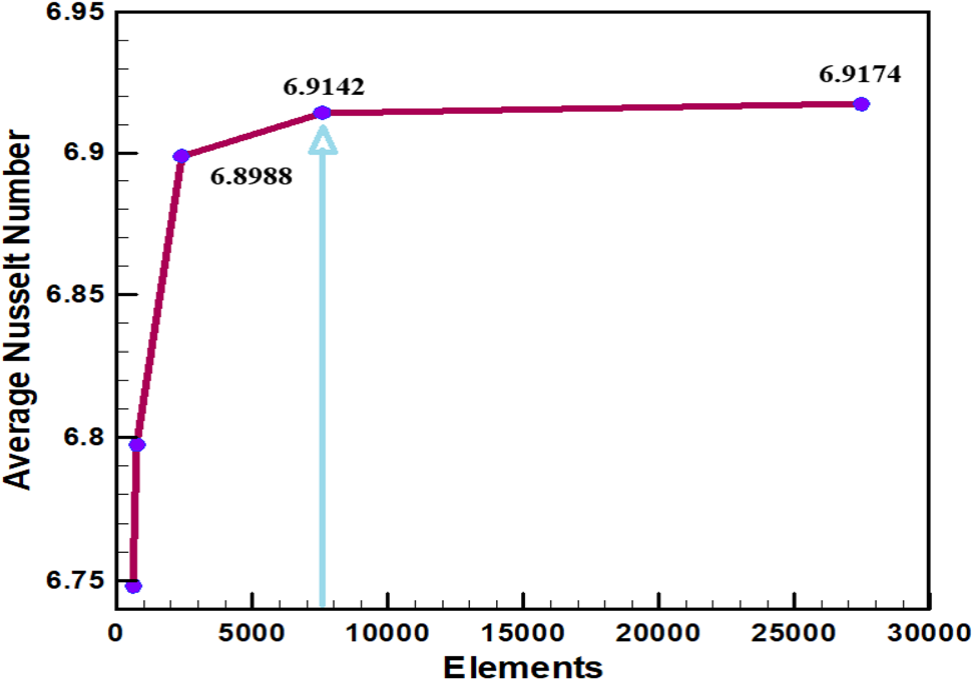
Grid measurement for various elements.

**Figure 3:**
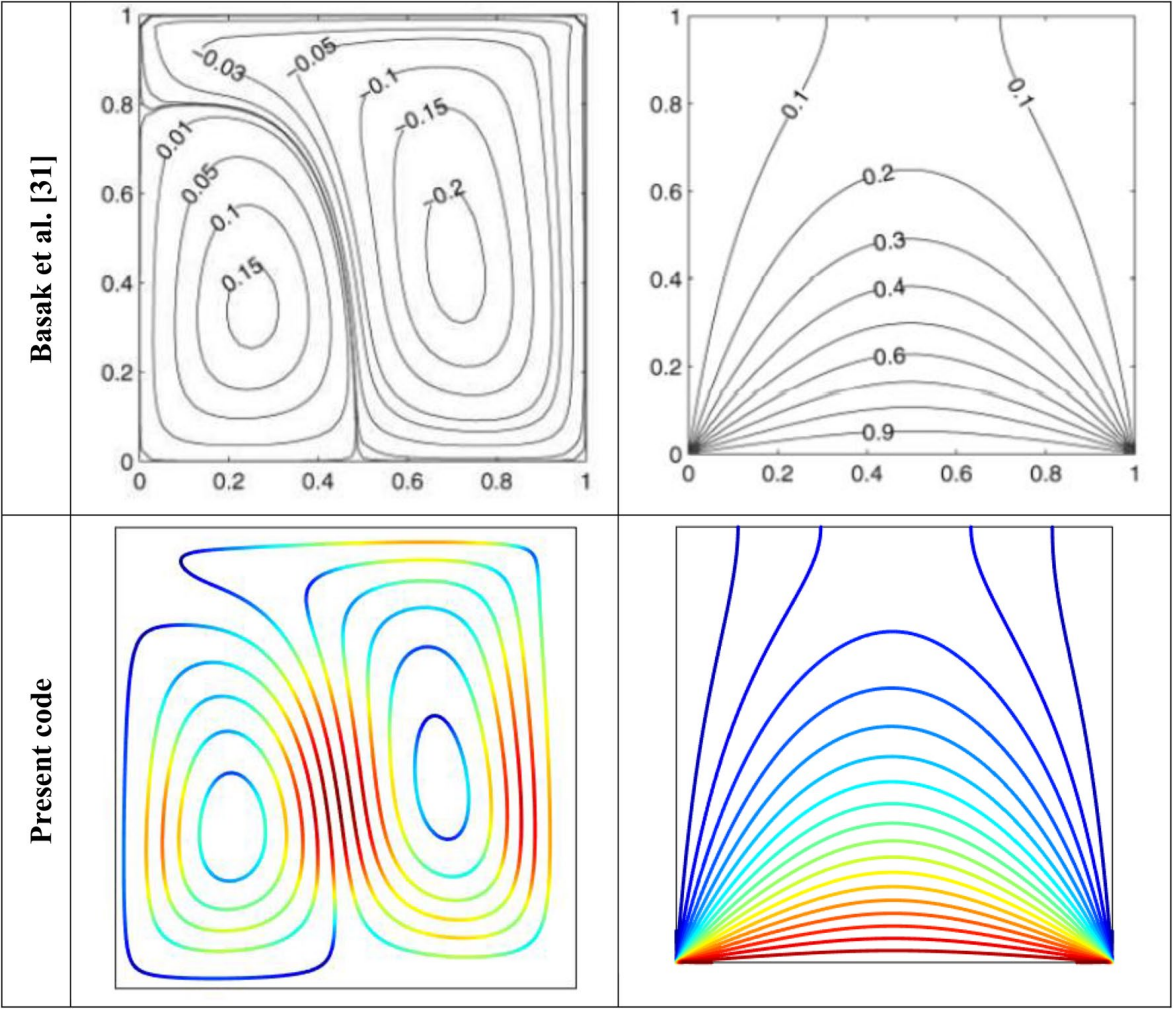
Parallelism of streamlines and isotherms obtained by current code and Basak et al*.*^33^.

To describe the appropriate grid shape for the current investigation at *Ri* = 1, *Pr* = 0.71, *W* = 0.40 and λ = 3. The average Nusselt ($Nu_{av}$) number of rectangular surfaces is derived in Table 1 and illustrated in Fig. 2, indicating negligible variation in grid shapes throughout a grid refinement test with many mesh types: An optimal solution for the current test could be found with 7575 nodes and 14,726 sizes. Figure 3 shows the parallelism of streamlines and isotherms obtained by current code and Basak et al.^33^

**Table 1:** An enquiry into grid test at *Ri* = 1, $\lambda = 3$, *Pr* = 0.71, and *W* = 0.40.

Nodes Elements	611 1089	737 1329	2387 4517	**7575** **14,726**	27,469 54,138
*Nu*_*av*_	6.7482	6.7974	6.8988	6.9142	6.9174

Significant values are in bold.

**Code validation**: Our results, corresponding to Nu, have been compared with the available results and found to be in complete agreement with Basak et al.^33^, as presented in Table 2. Furthermore, our results perfectly match those of Basak et al.^33^ as displayed in Fig. 3. This provides us with immense confidence to proceed with our computations for various values of different parameters.

## Results and discussions

**Figure 4:**
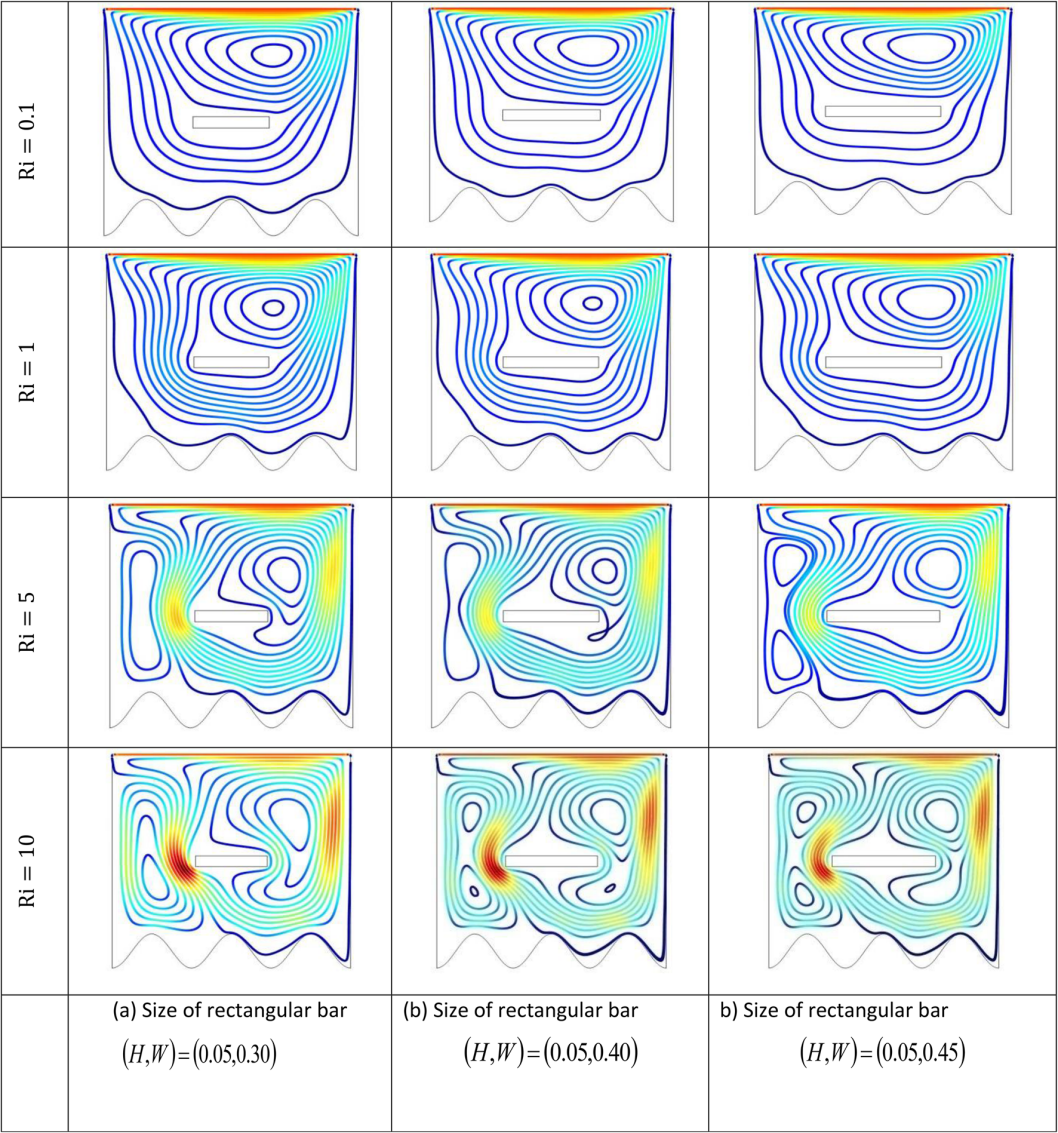
Effects of varying Richardson number on the streamlines for different size of rectangular heat source at Pr = 0.7, Ha = 0, Re = 100 and $\Delta = 0$.

**Figure 5:**
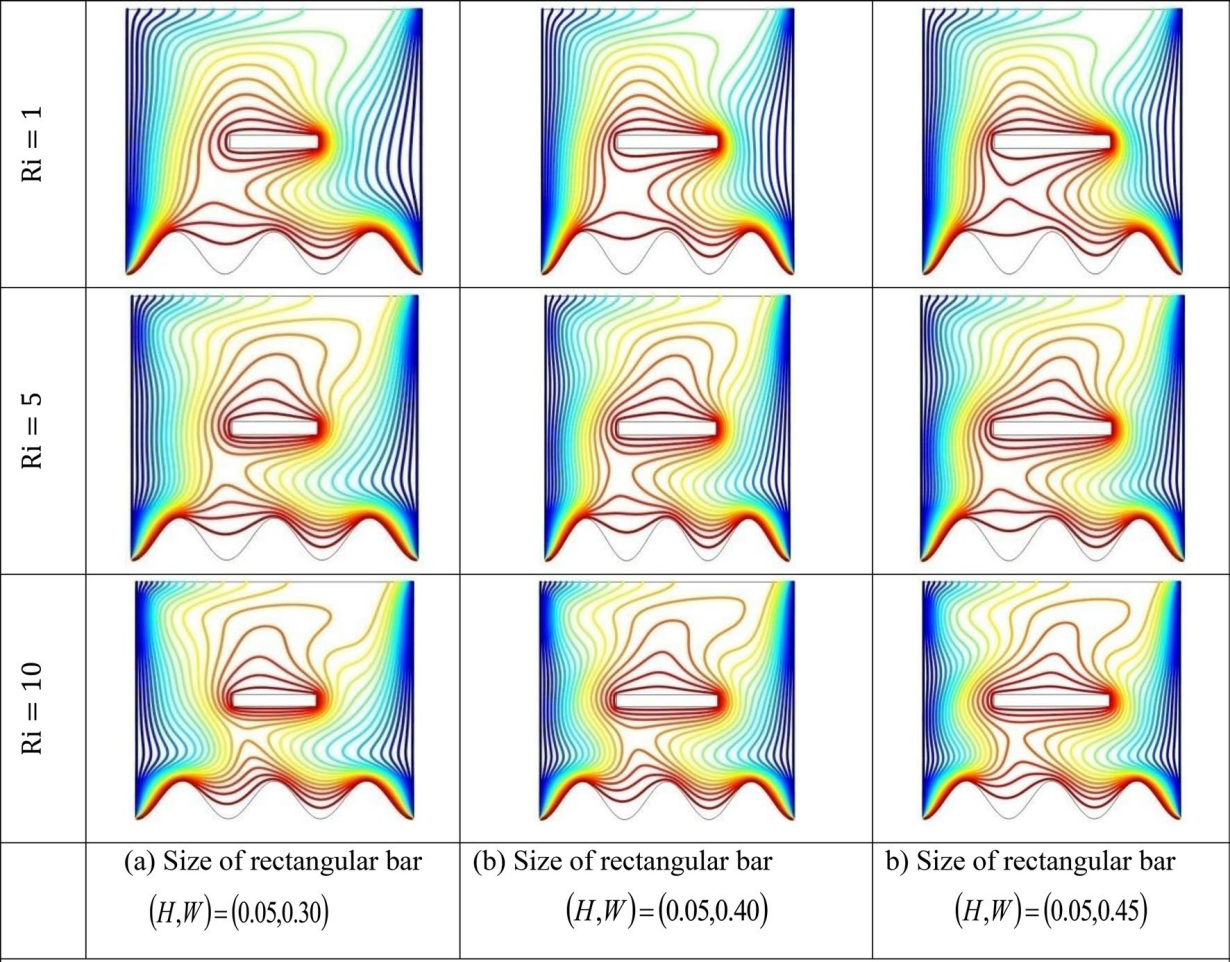
Effects of varying Richardson number on the isotherms for different size of rectangular heat source at *Pr* = 0.7, *Ha* = 0, *Re* = 100 and $\Delta = 0$.

**Figure 6:**
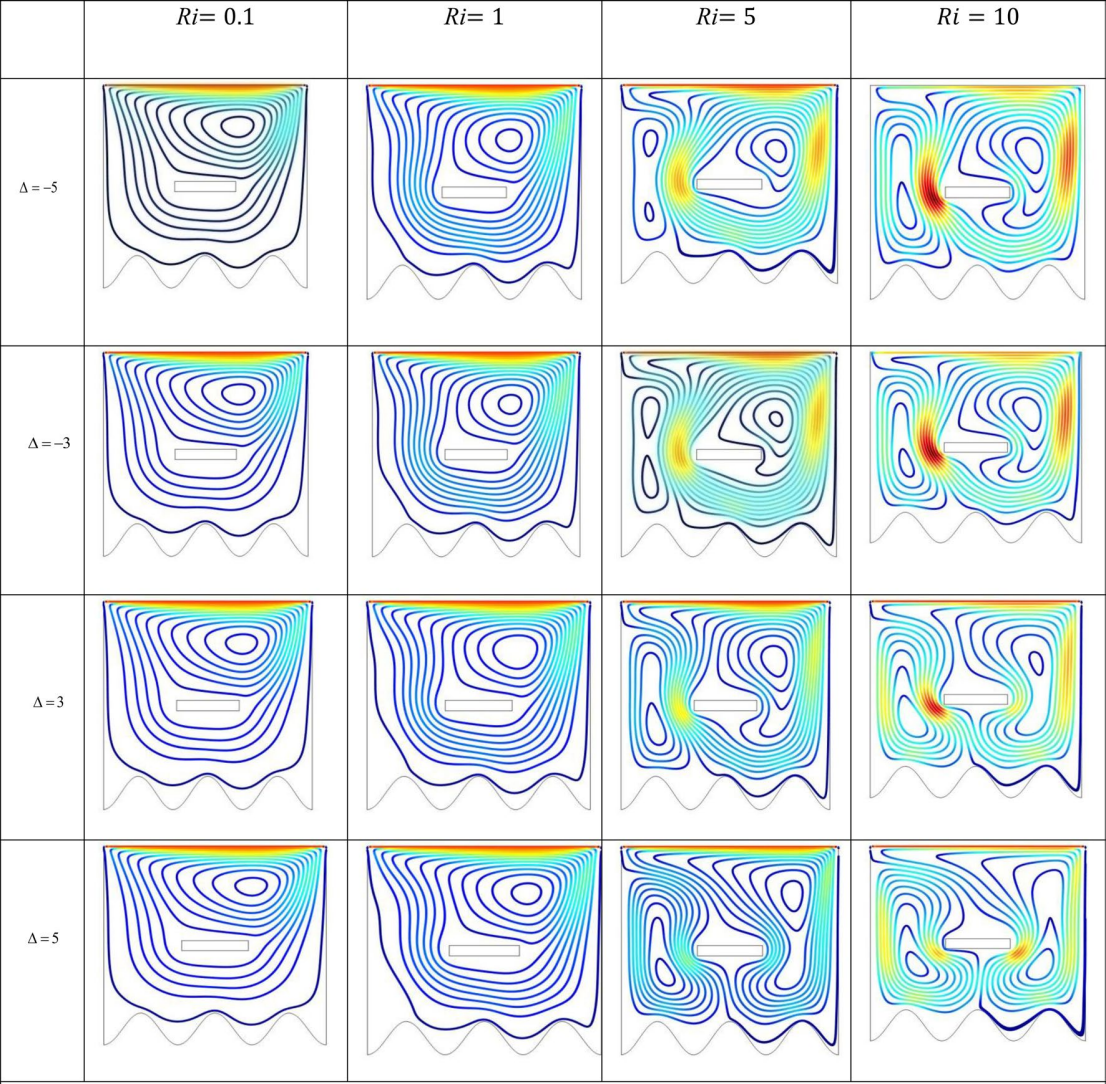
Effects of varying $\Delta$ on the streamlines for various *R*i at *Pr* = 0.7, *Ha* = 0, Re = 100, *H* = 0.05 and *W* = 0.30.

**Figure 7:**
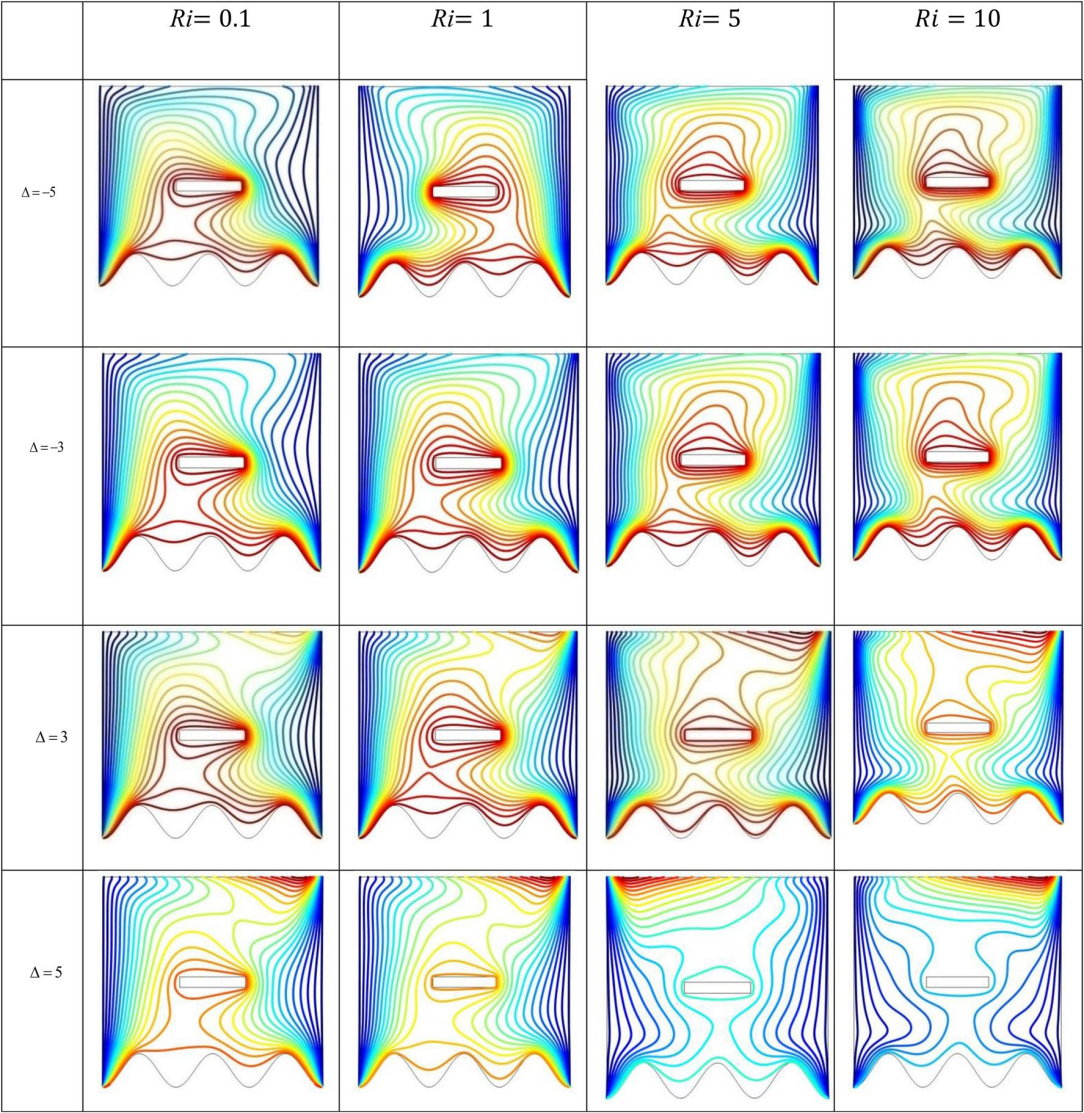
Effects of varying $\Delta$ on the isotherms for numerous Richardson number at *Pr* = 0.7, *Ha* = 0, *Re* = 100, *H* = 0.05 and *W* = 0.30.

**Figure 8:**
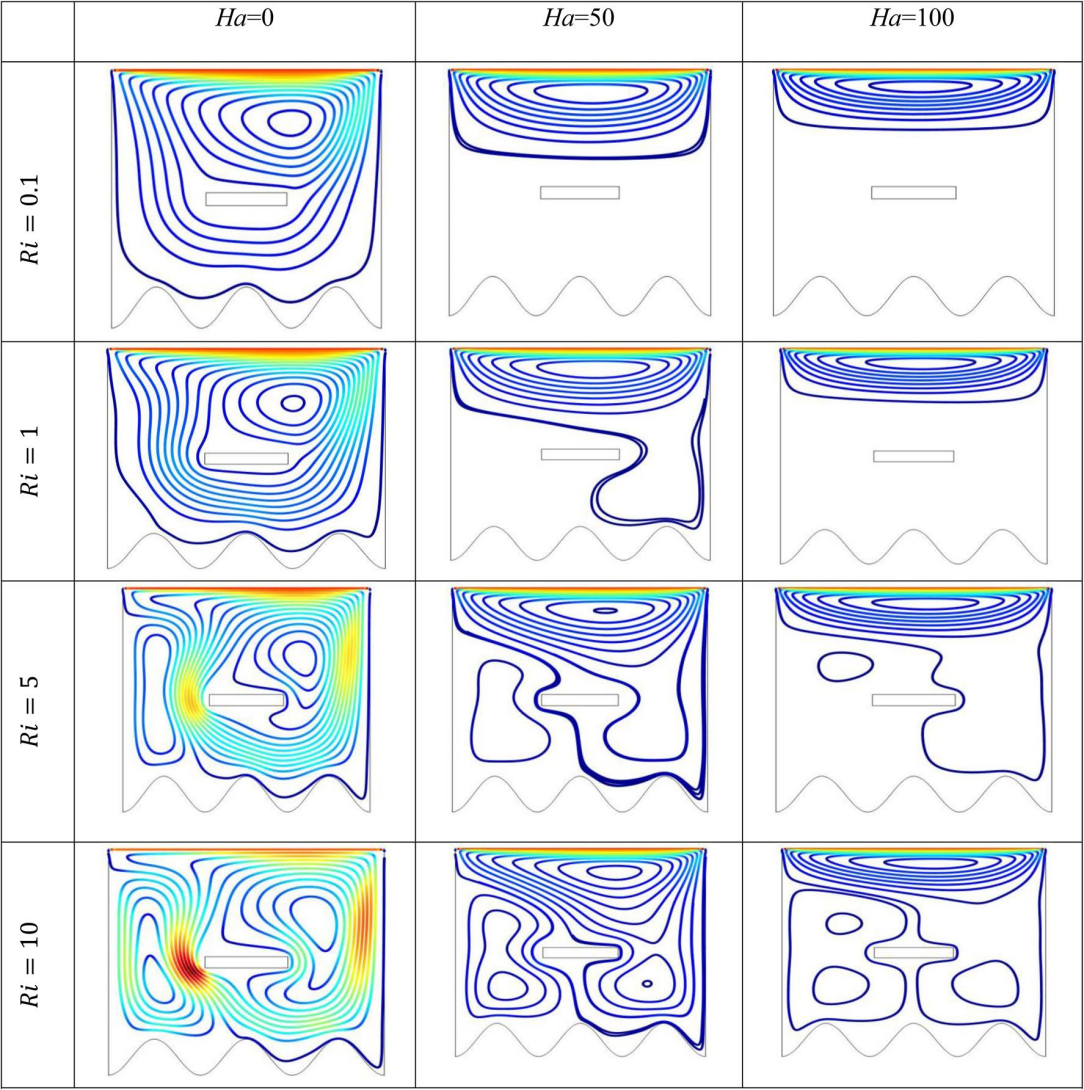
Effects of *Ha* on the streamlines for different size of *Ri* at *Pr* = 0.7, *Re* = 100 and $\Delta = 0$.

**Figure 9:**
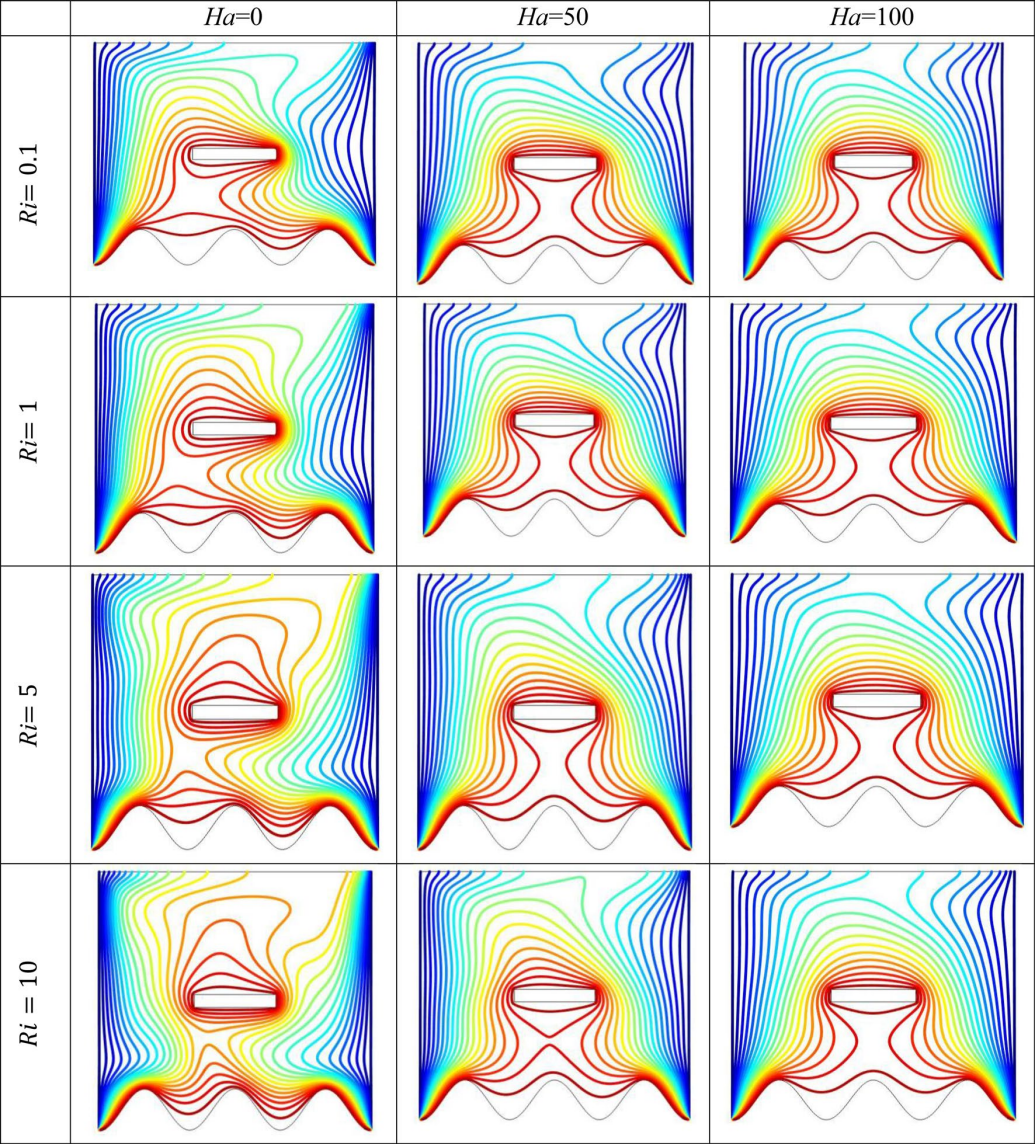
Effects of Ha on the streamlines for different size of *Ri* at *Pr* = 0.7, *Re* = 100 and $\Delta = 0$.

**Figure 10:**
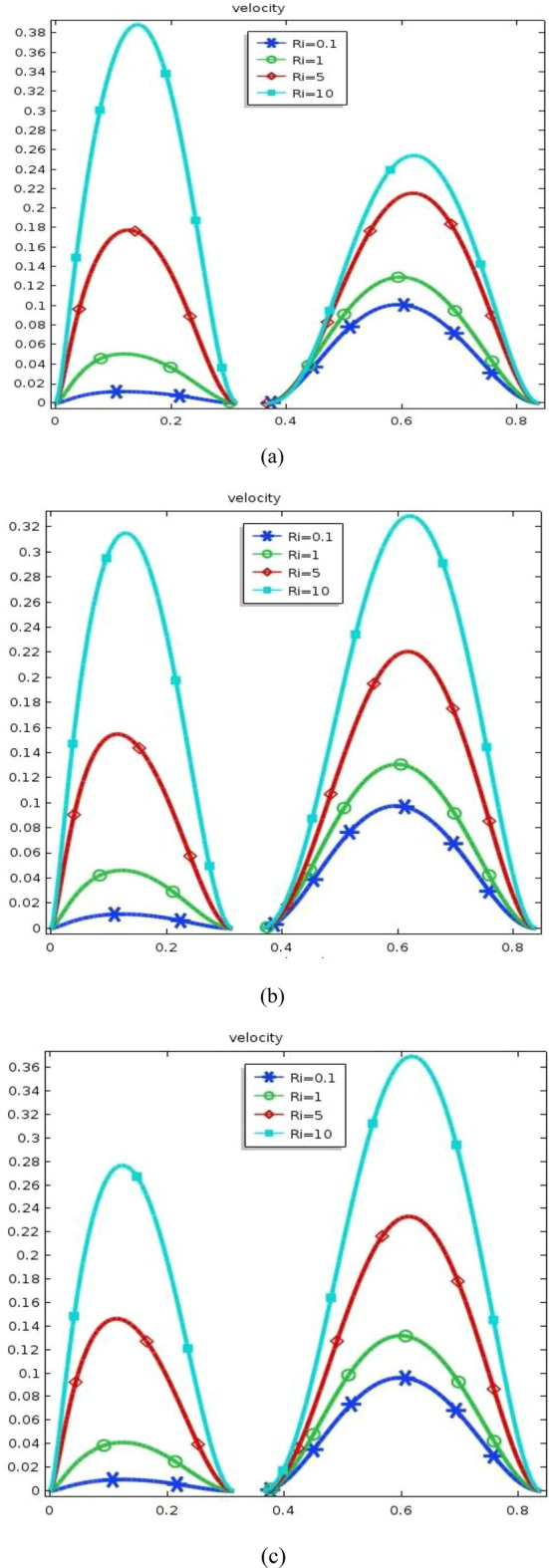
Velocity skeleton (**a**) *W* = 0.30, (**b**) *W* = 0.40, (**c**) *W* = 0.45 at *H* = 0.05, *Ha* = 0, and $\Delta = 0$.

**Figure 11:**
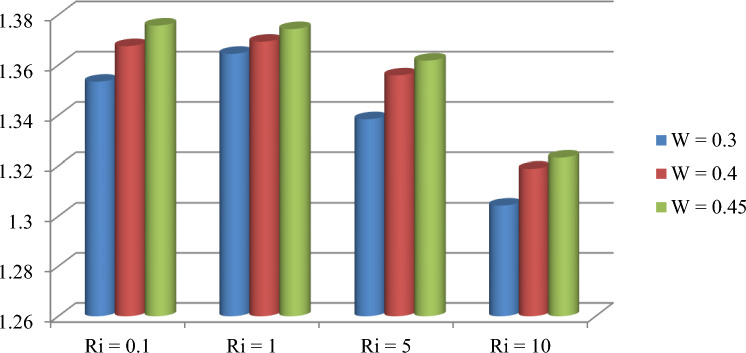
Rectangular heated source usefulness for different value of *W* while *Pr* = 0.7 , *H* = 0.05, *Ha* = 0, and $\Delta = 0$.

**Figure 12:**
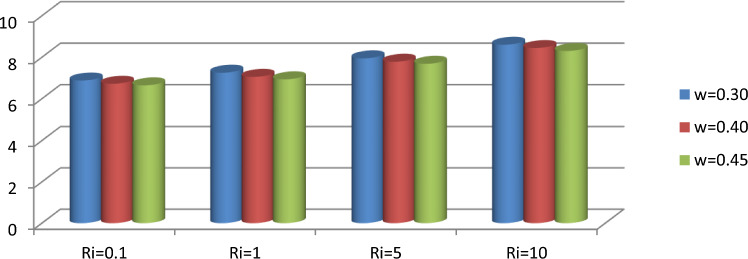
Average Nusselt numbers for several values of *Ha* and *Ri* with *Pr* = 0.7, *W* = , 0.45, *H* = 0.05 and $\Delta = 0$.

**Figure 13:**
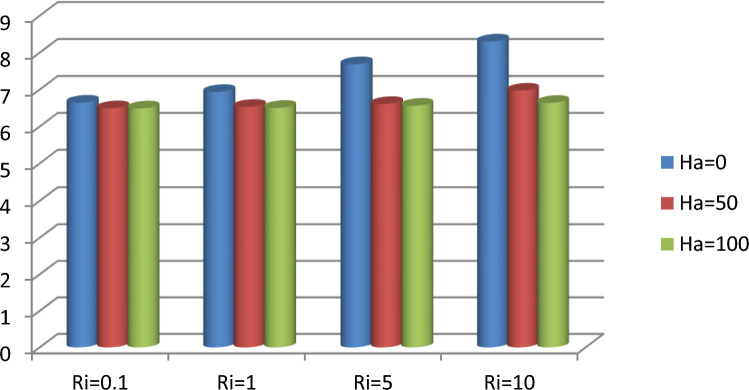
Average Nusselt Numbers for various values of *Ha* and *Ri* with *Pr* = 0.7, *W* = 0.45, *H* = 0.05 and $\Delta = 0$.

**Figure 14:**
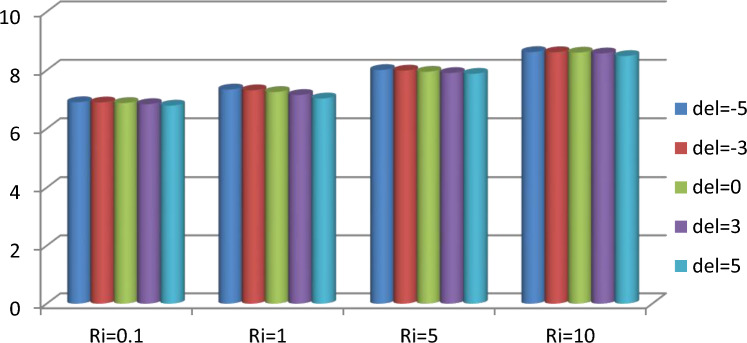
Average Nusselt Numbers for various values of $\Delta$ and *Ri* with *Pr* = 0.7, *W* = 0.30, *H* = 0.05 and Ha = 0.

The effect of an enclosed space rectangular heat source on MHD hybrid convective flow within a lid-driven enclosure with a heated waved bottom wall that has interior heat production, or absorption was examined in this section. Table 2 shows Nusselt number and heated wall bar width comparison. The current investigation has been speculated for several models of parameters. The following ranges of values are given Richardson number $Ri = 0.1 \le 1 \le 5 \le 10$,$\Pr = 0.7$, width of rectangular heated source $\left( {W = 0.30,0.40,0.45} \right)$,and height of heated source *H* = $0.05$,Ha = $0,50,100$, $\Delta = - 5, - 3,3,5$,Re = 100,Gr = $10^{3} - 10^{5}$, A(amplitude) = 0.08, $\lambda$(oscillation number) = 3. The outcomes are presented wavy shape cavity and then demonstrated graphically. Figure 4 displays varying Richardson numbers on the streamlines for several shapes of rectangular heat sources at *Pr* = 0.7, *Ha* = 0, *Re* = 100 and $\Delta = 0$. When *Ri* = 0.1 and 1 then the cavity's buoyancy strength is almost same with different rectangular heat source. Again, when *Ri* = 5 and 10 the cavity’s strength is significantly change and there is two eddies and with the width 0.30 of rectangular heated source and with increasing the width there exist vortices appear and it can be located left and right side on the closed space rectangular heated source. The real truth behind this was that the Richardson number and the rectangular length affected the flow area significantly influenced by the buoyancy force. The heat substitution of conduction is predominant viewed in Fig. 5. It is evident that a thermal boundary layer subsists near the heated surface (rectangular heat source and heated wavy bottom wall), and the thermal layer becomes unsubstantial for augmenting *Ri* and width of the rectangular heated source. The curving contour of isotherms is enhanced with growing *Ri* and *W,* and the heat lines are compressed to perpendicular sidewalls, which means the heat exchange via the convection.The impact of inner heat generation/creation or absorption coefficient $\Delta$ on the streamlines and isotherms as shown in Figs. 6 and 7. It has been shown from the Fig. 6, the streamlines almost stay unmovable for all values of $\Delta = - 5, - 3,3,5$ in the section of *Ri* = 0.1 and 1.Which shows that the free convective flow is completely enough to significantly affect the flow field. When *Ri* = 5 then a primary vortex is created and this becomes changes with the heat production or absorption parameters and the field of flow is changed. Therefore, the driving cover's flow has a greater impact than the buoyancy forces. The enhancing value of Gr raises the value of the Richardson number for a specific value of *Re*. When *Ri* = 10, the values of heat generation and absorption develops significant. If the values of $\Delta$ enhances, the flow field transforms into significant changing. The secondary minor eddies are the most prominent and increasing new cell with increasing $\Delta$ in magnitude and the minor vortices becomes symmetric with the major vortices. The Highest temperature occurs at the heated lower wall and heated rectangular edges in the absence of inner heat production or absorption coefficients. In contradict to the streamline, the isotherms within the wavy enclosure undergoes some changes within the effect of inner heat generation/creation or absorption. When *Ri* = 0.1, 1,5and 10, and the heat t is generated ($\Delta > 0$) then the maximum temperature is located to the principal region and the higher temperature area is spread out to the upper insulated wall. This is because the fluid is unable to reject any of its energy to the thermal wall above. This is due to the fact that the value of the Richardson number has been growing. In the event of inner absorption of heat, there is an opposing phenomenon that occurs, where the high temperature goes nearer to the hot basis of the structure. This happens because the base is becoming hotter. It is notable that the vertical walls, the shear force and buoyancy force both act in the identical direction and the energy is passes through the convection and the top wall the energy is passes through conduction. Subsequently the development of a robust thermal boundary layer in close proximity to the vertical walls has resulted in the conductive heat transmission system inside the enclosure becoming the dominant mode of energy transfer. The influence that a change in the Hartmann (*Ha*) number, has on the streamlines and isotherms found within the section with *Ri* = 0.1, 1, 5 and 10 is represented graphically in Figs. 8 and 9. When *Ri* = 0.1 and *Ha* is equal to zero, it is believed that the streamlines throughout the whole cavity area are dominated by a single main vortex but when *Ha* = 50, 100, the single primary vortex is situated to the upper of the rectangular heated surface. There are same significant changes for *Ri* = 1 and with increasing Hartmann number. When Ri = 5, 10 and *Ha* = 0, the cavity streamlines divided two vortices situated vertical sides of the rectangular bar, with increasing *Ha* the flow strength decreases gradually. Since we say that the flow strength reduces with higher Hartmann number. When the Hartmann number is not present the isotherms lines asymmetric and heat transmission are bounded by conduction and convection. In the presence of Hartmann number, the isotherm lines becomes symmetric with enhancing values of Hartmann number and Heat transmission is conduction mode only. Impression of Richardson (*Ri*) number on velocity profiles adjoining the parallel center-line of the rectangular enclosure at a few rectangular sources of heat (a) *W* = 0.30, (b) *W* = 0.40, (c) *W* = 0.4, at H = 0.05, *Pr* = 0.7 and $\Delta = 0$ are exposed in Fig. 10. It could be noticed that for low Richardson values velocity segments carry more trivial changes, but at the high Richardson number velocity drawings cause more significant changes. Rising Richardson numbers result in increased velocities. The effectiveness of a changeable rectangular heated source is a parameter value that measures the enhancement of removing heat within a cavity when a rectangular heat source is used compared than one without a rectangle. While all other factors are held constant, this section plots the efficiency of a rectangular bar against the Rayleigh number over a range of widths (*W*) of the heat source. These are demonstrated in Table 3 and Fig. 11. The effectiveness of a rectangular heat source is found to gradually decline with rising Ri for a given rectangular source size. Table 3 shows that the highest and lowest efficiency values are 1.375741 and 1.304265, respectively. The average Nuselt ($Nu_{av}$) number to the heated surface versus Richardson number with dissimilar rectangular heated sources (*W* = 0.30, 0.4, and *H* = 0.05), while the other variables remain unchangeable. These are presented in Table 4 and Fig. 12. It has been shown from the figure that the average Nusselt ($Nu_{av}$) number decreases calmly with the enhancing Ri for the various shape of rectangular source. Moreover, as the heat source of the rectangle increases the average Nusselt ($Nu_{av}$) number reduces with a heated wall for whole Richardson number of the enclosures. The average Nusselt ($Nu_{av}$) number to heated surface versus Richardson number with differing Hartmann number with rectangular heat sources width and height is *W* = 0.45, *H* = 0.05, while the usage of the residual parameters is static. These are demonstrated in Table 5 and Fig. 13. It is shown from the figure that the average Nusselt ($Nu_{av}$) number increasing calmly with the rising Ri for the various shapes of rectangular source, but with increasing Ha the average Nusselt ($Nu_{av}$)number reducing. Again, the average Nuselt ($Nu_{av}$) number along the heated wall versus Richardson number with varying $\Delta$ with rectangle shape heat source width and height is *W* = 0.30, and *H* = 0.05, while the usage of the residual parameters is static. Table 6 and Fig. 14 both illustrate these results. It is shown from the figure that the average Nusselt ($Nu_{av}$) number increasing calmly with the augmenting Ri for the various shapes of rectangular source for all values of $\Delta$. When heat is absorbed d ($\Delta < 0$) the $Nu_{av}$ is augmented and when heat is generated ($\Delta > 0$) the $Nu_{av}$ is decreased.

**Table 2:** Nusselt number and heated wall bar width comparison.

Ra	Average Nusselt ($Nu_{av}$) number					
	W = 0.20		W = 0.25		W = 0.30	
$10^{4}$	1.108865	2.4296	1.122605	2.4529	1.132565	2.4679
$10^{5}$	1.123183	2.5724	1.132975	2.6089	1.147419	2.6407
$10^{6}$	1.147287	2.6686	1.160168	2.7166	1.172883	2.7430
	Asad et al.^30^	Present code	Asad et al.^30^	Present code	Asad et al.^30^	Present code

**Table 3:** Bar effectiveness.

*Ri*	Bar effectiveness		
	*W* = 0.30	*W* = 0.40	*W* = 0.45
0.1	1.353375	1.36755	1.375741
1	1.364484	1.369317	1.374319
5	1.338449	1.3559	1.361787
10	1.304265	1.318816	1.323201

**Table 4:** Average Nusselt numbers for heated wavy wall for variation of rectangular bar.

*Ri*	Average Nusselt numbers		
	*W* = 0.30	*W* = 0.40	*W* = 0.45
0.1	6.8779	6.722	6.646
1	7.2556	7.0542	6.9359
5	7.9478	7.7845	7.6856
10	8.6039	8.4452	8.3033

**Table 5:** Average Nusselt numbers for heated wavy wall with variation of Ha.

*Ri*	Average Nusselt numbers		
	*Ha* = 0	*Ha* = 50	*Ha* = 100
0.1	6.6460	6.4935	6.4901
1	6.9358	6.5326	6.5033
5	7.6856	6.6094	6.5628
10	8.3033	6.9716	6.6397

**Table 6:** Average Nusselt numbers for heated wavy wall for variation of $\Delta$.

*Ri*	Average Nusselt numbers				
	$\Delta = - 5$	$\Delta = - 3$	$\Delta = 0$	$\Delta = 3$	$\Delta = 5$
0.1	6.9060	6.8977	6.8779	6.8393	6.7890
1	7.3398	7.3138	7.2556	7.1532	7.0341
5	8.0081	7.9875	7.9478	7.9047	7.8788
10	8.6226	8.6168	8.6039	8.5751	8.4877

### Bar effectiveness

According to Asad et al.^30^, bar efficacy is a changeable that quantifies the heat transformation augmentation that occurs in a wavy shape enclosure when the bar is compared to a scenario where there is no bar:

$$E_{f} = \frac{{Q_{bar} }}{{Q_{without\,\,bar} }}$$

## Conclusion

This study extensively investigated the effects of a heated rectangular source within an enclosed space on MHD hybrid convection flow. The space was characterized by a lid-driven chamber featuring a wavy bottom surface that underwent internal heat generation, creation, or absorption. The study specifically considered a Prandtl number of 0.7. Utilizing the Galerkin Finite Element (FE) technique, the governing equations were effectively solved. The primary emphasis of this research lay in analyzing the factors that exerted influence over heat distribution and the flow field. Notably, the study unveiled a strong correlation between the heat transfer process and flow behaviors occurring within the wavy cavity and the Hartmann (*Ha* = 0, 50, 100) number, Richardson (*Ri* = 0.1, 1, 5, 10) number, the size of the heated rectangular bar [H.W. = (0.05,0.30), (0.05,0.40), (0.05,0.45)]*,* and heat generation or absorption coefficient ($\Delta = - 3, - 5,3,5$). The perceived numerical outcomes can be shortened as follows:

- The change in the Richardson number appears to have had a considerable effect on the flow area within the wavy cavity.
- The rise in Richardson number also had an effect on the strength of the streamline field and Isotherms.
- Increasing the Richardson number for the size of all rectangular heat sources improves the efficiency of a rectangular heated source. The efficiency of a rectangular heated source is also stated as increasing with the width of the rectangular heated source.
- With a hot wall, the $Nu_{av}$ (average Nusselt) number drops as the rectangular heat source grows for all Richardson numbers of the enclosures. As the $Nu_{av}$ number declines, the reversal behaviour indicated by the rectangular heat source rises.
- The existence of Hartmann numbers has a major impression on the flow behaviour and heat transference qualities inside the cavity section. The flow's energy drops, and the isotherms become symmetrical as *Ha* increases in value. The $Nu_{av}$ number decreased as the Hartmann number increased.
- The existence of the influence of internal heat generation lowered the $Nu_{av}$ number substantially. The existence of internal heat absorption, which raises the $Nu_{av}$ number for all Richardson numbers, indicated accurate reversal behaviour.

In the near future, the research might be expanded to include unsteady instances, natural convection, porous media, nano-fluids, and three-dimensional analysis.

## Data Availability

All data generated or analyzed during this study are included in this manuscript and there is no permission from a third party.

## List of symbols

$$A$$: Wave amplitude
$$B_{0}$$: Magnetic field
$C_{p}$: Specific heat coefficient, ${\text{J}}{\kern 1pt} \;{\text{kg}}^{ - 1} \;{\kern 1pt} {\text{k}}^{ - 1}$
$g$: Acceleration due to gravity, ${\text{m}}\;{\text{s}}^{ - 2}$
$h *$: Heat transference coefficient
$k$: Parameter of thermal conductivity, ${\text{W}}\,{\text{m}}^{ - 1} \,{\text{k}}^{ - 1}$
$L$: Height and width of the enclosure
$Nu_{av}$: Average Nusselt number
$Nu_{L}$: Local Nusselt number
$$Ha$$: Hartmann number, $$\sqrt {\frac{{\sigma B_{0}^{2} L^{2} }}{\mu }}$$
$$Gr$$: Grashof number,$$\frac{{g\beta \Delta TL^{3} }}{{\upsilon^{2} }}$$
$$\Pr$$: Prandlt number, $$\frac{\upsilon }{\alpha }$$
$${\text{Re}}$$: Reynolds number,$$\frac{{U_{0} L}}{\upsilon }$$
$\Delta$: Heat generation or absorption coefficient, $\frac{{Q_{0} L^{2} }}{{\alpha \rho C_{p} }}$
$U\,,V$: Dimensionless velocity in $X$ and $Y$ direction respectively

## Greek symbols

$\alpha$: Thermal diffusivity coefficient, ${\text{m}}^{2} \;{\text{s}}^{ - 1}$
$\beta$: Thermal expansion parameter, ${\text{k}}^{ - 1}$
$$\lambda$$: Oscillations number
$$\rho$$: Fluid density, $${\text{kg}}\;{\text{m}}^{ - 3}$$
$\theta$: Non-dimensional temperature
$$\mu$$: Dynamic viscosity, $${\text{kg}}\;{\text{m}}^{2} \;{\text{s}}^{ - 1}$$
$$\upsilon$$: Kinematic viscosity,$${\text{m}}^{2} \;{\text{s}}^{ - 1}$$
$$\psi$$: Stream function

## Subscripts

$$av$$: Average/mean
$$c$$: Cool
$$h$$: Hot
